# On a Model of Pattern Regeneration Based on Cell Memory

**DOI:** 10.1371/journal.pone.0118091

**Published:** 2015-02-19

**Authors:** Nikolai Bessonov, Michael Levin, Nadya Morozova, Natalia Reinberg, Alen Tosenberger, Vitaly Volpert

**Affiliations:** 1 Institute of Problems of Mechanical Engineering, Russian Academy of Sciences, 199178 Saint Petersburg, Russia; 2 Department of Biology, Tufts Center for Regenerative & Developmental Biology, Tufts University, Medford, MA 02155, USA; 3 Laboratoire Epigénétique et Cancer, CNRS FRE 3377, CEA, 91191 Saclay, France; 4 Institut des Hautes Etudes Scientiques, 91440 Bures-sur-Yvette, France; 5 Institut Camille Jordan, UMR 5208 CNRS, University Lyon 1, 69622 Villeurbanne, France; Centro Cardiologico Monzino, ITALY

## Abstract

We present here a new model of the cellular dynamics that enable regeneration of complex biological morphologies. Biological cell structures are considered as an ensemble of mathematical points on the plane. Each cell produces a signal which propagates in space and is received by other cells. The total signal received by each cell forms a signal distribution defined on the cell structure. This distribution characterizes the geometry of the cell structure. If a part of this structure is removed, the remaining cells have two signals. They keep the value of the signal which they had before the amputation (memory), and they receive a new signal produced after the amputation. Regeneration of the cell structure is stimulated by the difference between the old and the new signals. It is stopped when the two signals coincide. The algorithm of regeneration contains certain rules which are essential for its functioning, being the first quantitative model of cellular memory that implements regeneration of complex patterns to a specific target morphology. Correct regeneration depends on the form and the size of the cell structure, as well as on some parameters of regeneration.

## Introduction

Numerous species are able to restore complex body organs after amputation [1]. For example, planaria can regenerate their entire body from a small fragment [2], and axolotls can restore limbs, spinal cord, jaws eyes, hearts, and portions of their brain [3]. Learning to control this process is the key to transformative applications in biomedicine [4, 5], as well as pattern control of embryogenesis (with applications to birth defects). While the field is rapidly accumulating high-resolution data on the genetic networks and molecular components necessary for this process [6], fundamental insight into complex shape homeostasis is lacking. This is related to a dearth of testable models explaining the signaling dynamics sufficient to explain how the correct pattern is regenerated and how growth ceases when the proper anatomy is restored. One of the main open questions is whether the regenerating organism uses only the information available at each particular moment of time or whether it can also use information about its former state—a pattern memory [7, 8]. In the first case, in order to reproduce complex forms and different organs, we need to deal with pattern formation and emergence of forms. This is often modeled via Turing structures and other mechanisms of pattern formation and self-organisation [9, 10, 11, 12, 13, 14, 15]. The process of regeneration then depends on the regeneration of patterns. If this pattern corresponds to a stationary solution of a reaction-diffusion model, then amputation can be considered as a perturbation of this stationary solution and regeneration corresponds to decay and disappearance of this perturbation.

In contrast, the organism may keep information about its original state and restores pattern after damage by minimizing the difference between the current state and the original state (a target morphology model) [5, 16, 17]. At this time, no quantitative model of target morphology during pattern formation exists.

In this work we suggest a mathematical model based on the assumption that regeneration uses the memory of the organism about its original state. This model provides a proof-of-principle of a mechanistic model that implements patterning towards an encoded target morphology memory, and illustrates a system to formulate the assumptions necessary for regeneration of cellular structures in mathematical models.

The scheme presented below is based on the assumption that there are cells that can preserve state information for some time (memory cells). This is realistic since a wide variety of somatic cell types, not only neurons, have been shown to exhibit memory [18, 19, 20, 21, 22, 23]. We model it as follows. Suppose that a cell receives some signal *u* with a given intensity *u* = u* (this could be concentration of a signaling molecule, or a bioelectric signal [24, 25], or any other kind). After some time, when the signal disappears or changes its value, the information about the old value u* is preserved in the cell. Moreover, we presume that the cell can measure the difference between the old value and the current (new) value, u*−*u*, and produce another signal *z* with a rate proportional to this difference.

There are various kinds of cells that exhibit memory (neural cells, lymphocytes, plants, bacteria) via different mechanisms. These assumptions thus do not contradict available biological information but it is not yet known whether memory processes operate during tissue regeneration.

We suggest a possible mechanism which can provide these properties. Let the signal *u* correspond to the concentration of some substance *A* in a volume bounded by a membrane. Its value in this volume equals u* and is kept fixed. At the other side of the membrane *u* = 0. Its flux through the membrane is proportional to the difference of the values, that is to u*. This flux leads to the appearance of stable objects *B* (e.g., ion channels, molecules) which participate in processing of *A* (transport, consumption, reaction). The quantity of *B* is proportional to the flux of *A*, that is to u*. If *u* decreases, then some part of *B* is liberated from processing of *A* and becomes involved in other reactions. They lead to production of another signal *z*. This mechanism keeps memory either about the maximal value = u* or about the value which is kept during some time sufficient to create *B*.

We note that the mechanisms which allow cells to determine and to preserve the maximal signal were suggested and discussed by [13].

The signals can be transported either from cell to cell proportionally to the difference of concentrations or by diffusion through the extracellular matrix.

The implementation of the model is based on the method of cellular automata where cells are located at the nodes of a square grid. This method is a particular case of more general cellular nonlinear networks. Cells communicate with each other due to the exchange of signals and they can divide according to some algorithm described in the next section. Cellular automata are widely used to model growth of biological tissues (see [28], and the references therein). Tissue regeneration is studied for example in a more general Neuronal Organism Evolution model [26, 27]. The main objective of this work is to develop a minimal model of regeneration based on plausible biological assumptions, which would constructively explain the exact regeneration of an arbitrary (within limit) amputated part of the organism.

## Model of regeneration

### Signal distribution

Consider a 2D domain *D* filled by cells. Each cell produces a signal *u* which spreads in space. Its intensity decays with distance as some function *f*(*d*). If the distance between cell *i* (*C*_*i*_) and cell *j* (*C*_*j*_) is *d*_*ij*_, then *C*_*j*_ receives signal *f*(*d*_*ij*_) from *C*_*i*_. We will assume here that each cell produces the same signal. Therefore *C*_*i*_ receives from *C*_*j*_ a signal of the same intensity *f*(*d*_*ij*_). For each cell *C*_*i*_ we can count the total signal which it receives from other cells

$$\begin{array}{c} u_{i}=\sum_{j\neq i}f\left(d_{ij}\right). \end{array}$$ (1)

We will use also the notation *u*(*x*) assuming that *x* belong to the domain *D*, *u*_*i*_ = *u*(*x*_*i*_), where *x*_*i*_ is the coordinate of the *i*th cell.

Clearly, cells located in different parts of the domain will receive different signals. For example, a cell located at the boundary receives signals only from one side and the value of the signal can be less than for a cell inside the domain. Therefore the distribution *u*(*x*) represents some information about the geometry of the domain.

**Fig 1:**
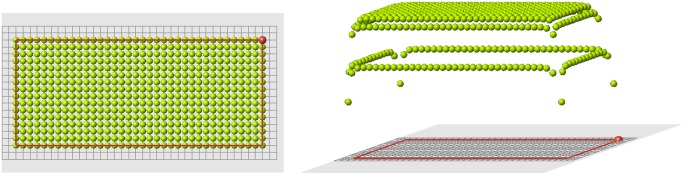
Rectangular domain filled by cells (left). The value of the signal in each cell (right), *f*(*d*) = 1/*d* ^2^.

**Fig 2:**
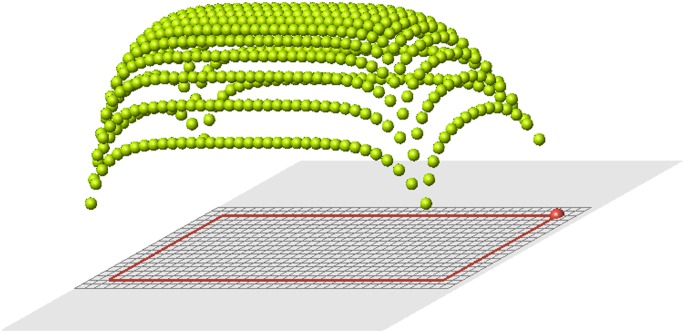
Signal distribution for *f*(*d*) = 1/*d* ^0.5^.

Let us consider some examples. Signal distribution for a rectangular domain is shown in Fig. 1. Here *f*(*d*) = 1/*d* ^2^. The value *u*(*x*) at the boundary is less than inside the domain, and the value at the corners is less than in other points of the boundary. The signal distribution depends on the function *f*(*d*) (Fig. 2).

### Reduction of the domain

**Fig 3:**
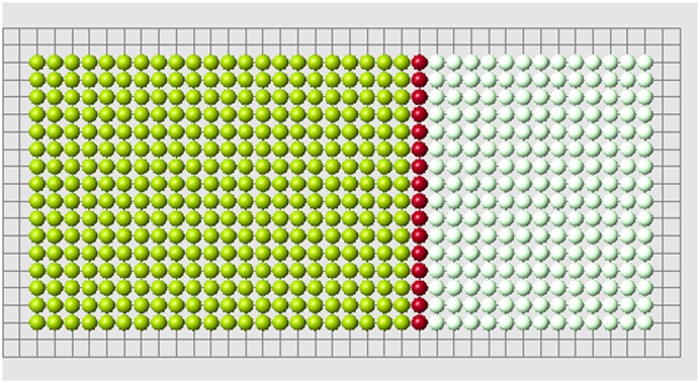
A set of cells is removed. White cells show places of removed cells. Red cells are the remaining cells which are at the boundary with the removed cells (blastema).

Suppose that a part of cells is removed (white cells in Fig. 3). The remaining cells will be called control cells. We can define two signals in control cells: the old signal

$$\begin{array}{c} u_{i}^{*}=\sum_{j\neq i,j\in I_{0}}f\left(d_{ij}\right),\;\;\;\;i\in I_{c}, \end{array}$$

where *I*_0_ is the set of cells in the original cell structure, *I*_*c*_ is the set of control cells, and the new signal

$$\begin{array}{c} u_{i}=\sum_{j\neq i,j\in I_{c}}f\left(d_{ij}\right),\;\;\;\;i\in I_{c}. \end{array}$$

**Fig 4:**
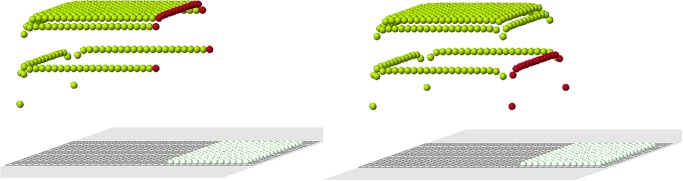
Signal distribution in control cells for the complete structure (left) and for the reduced structure (right).

The old signal is measured in control cells from all cells in the original structure before amputation. The new signal is measured also in control cells which they receive from remaining (control) cells after amputation. These two signal distributions are different, and that difference is clearly seen near the cut (Fig. 4).

### Updating cells: analytical examples

When a part of the organism is removed, the signal distribution in the remaining cells, which we call the control cells, is changed. The the question is then how to restore the cell configuration with the initial signal distribution. Remaining cells near the cut will start to divide. New cells will contribute to the signal distribution and will continue to divide filling available places in their neighborhood. This growing structure is regulated by the signal distribution in control cells and will converge to the initial structure.

We will begin with some simple examples that admit analytical solutions. Consider three cells, *A*, *B* and *C*. Let us remove the cell *C* and discuss the algorithm of its restoration. Denote by *d*_*AB*_, *d*_*BC*_ and *d*_*AC*_ the distances between the corresponding cells. Before removal the cell *C*, the signals received by cells *A* and *B* are as follows:

$$\begin{array}{c} u_{A}^{*}=f\left(d_{AB}\right)+f\left(d_{AC}\right),\;\;\;u_{B}^{*}=f\left(d_{AB}\right)+f\left(d_{BC}\right). \end{array}$$

When this cell is removed, they become

$$\begin{array}{c} u_{A}=f\left(d_{AB}\right),\;\;\;u_{B}=f\left(d_{AB}\right). \end{array}$$

We put cell *C* in an arbitrary place and denote by ${\tilde{d}}_{BC}$ and ${\tilde{d}}_{AC}$ its distances to the cells *A* and *B*. The new signals received by these cells are now

$$\begin{array}{c} u_{A}=f\left(d_{AB}\right)+f\left({\tilde{d}}_{AC}\right),\;\;\;u_{B}=f\left(d_{AB}\right)+f\left({\tilde{d}}_{BC}\right). \end{array}$$

We need to choose the position of cell *C* in such a way that $u_{A}=u_{A}^{*}$, $u_{B}=u_{B}^{*}$. These equalities are satisfied if

$$\begin{array}{c} f\left({\tilde{d}}_{AC}\right)=f\left(d_{AC}\right),\;\;\;f\left({\tilde{d}}_{BC}\right)=f\left(d_{BC}\right). \end{array}$$

Since *f*(*d*) is a monotonically decreasing function, we conclude that ${\tilde{d}}_{AC}=d_{AC}$, ${\tilde{d}}_{BC}=d_{BC}$. These equalities determine two circles on the plane. One of them is around point *A* with radius *d*_*AC*_, another one is around point *B* with radius *d*_*BC*_. They intersect in two points. One of them corresponds to the initial position of cell *C*, and another one is symmetric with respect to the line *AB*.

Thus, in the case of three cells, one removed cell can be restored by a simple algorithm described above. A similar approach is applicable for any initial number of cells *n* if only one cell is removed. It should be noted that in general *n*−1 circles may not have a point of intersection. However in the problem considered here their intersection is guaranteed by the initial configuration.

If more than one cells is removed, the problem of their restoration does not have a simple analytical solution except for the case where all cells are located at the same straight line. The existence of solutions of the restoration problem is provided by the initial configuration. So the question is how to converge to this configuration adding new cells one after another. A possible algorithm is suggested in the next section.

### Algorithm of regeneration

We will use the difference between the signals $u_{i}^{*}$ and *u*_*i*_, *i* ∈ *I*_*c*_ in order to restore the initial form. The issue here is the algorithm of decision-making that determines where to put new cells. We will add new cells one after another and denote the set of cells in the process of regeneration by *I*(*t*). Here *t* is discrete time, *t* = *t* ^*n*^, where at each next time step we add one cell. Let us introduce the signal

$$\begin{array}{c} u_{i}\left(t\right)=\sum_{j\neq i,j\in I(t)}f\left(d_{ij}\right),\;\;\;\;i\in I_{c}. \end{array}$$

This means that we measure the total signal from old and new cells in the control cells. The purpose is to restore a structure for which

$$\begin{array}{c} u_{i}\left(T\right)=u_{i}^{*},\;\;\;\;i\in I_{c} \end{array}$$

for some time *t* = *T*.

We suggest an algorithm for the placement of new cells determined by the following three conditions:

1. All cells are placed in the nodes of the square grid. Each cell has 8 neighbors, 4 of them with a common side and 4 other cell with a common diagonal. Each new cell is placed in such a way that among its neighbors there is at least one cells from the cut (blastema) or another new cell. This condition provides continuity of growth of the regenerating domain, beginning from the location of the cut.

2. When we add a new cell we recalculate the signal in every control cell. The new signal should be less than or equal to the old signal,

$$\begin{array}{c} u_{i}\left(t\right)\leq u_{i}^{*},\;\;\;\;i\in I_{c}. \end{array}$$

In numerical simulations this condition should be satisfied with certain accuracy (Section 3.1).

3a. Let us introduce total signals:

$$\begin{array}{c} S^{*}=\sum_{i\in I_{c}}u_{i}^{*},\;\;\;\;S\left(t\right)=\sum_{i\in I_{c}}u_{i}\left(t\right). \end{array}$$

Among all cells, which satisfy conditions 1 and 2, at each time step we choose that cell for which the difference S* − *S*(*t*) is minimal.

Let us illustrate this condition with the following example. Suppose that there are only two control cells *A* and *B*, and we choose where to place a new cell *C*. For each possible position of cell *C*, we measure the signal *S*(*AC*) received by cell *A* from cell *C* and the signal *S*(*BC*) received by cell *B* from cell *C*. We put cell *C* in the place where the sum of these two signals is maximal.

3b. Each control cell produces a signal proportional to the difference $u_{i}^{*}-u_{i}(t)$. This signal spreads in space and stimulates appearance of new cells. We choose the cell, which satisfies conditions 1 and 2, and where the value

$$\begin{array}{c} z_{k}=\sum_{i\in I_{c}}f\left(d_{ik}\right)\left(u_{i}^{*}-u_{i}\left(t\right)\right) \end{array}$$

is maximal. Here *k* belong to the set of cells, which satisfy conditions 1 and 2.

Conditions 3a and 3b give close results. In the first case, we choose the cell which adds the greatest signal to the control cells. In the second case, we choose the cell which gets the greatest signal from the control cells. The second algorithm is more biologically plausible.

**Fig 5:**
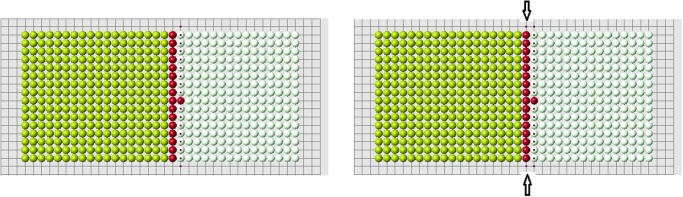
The choice of the first cell. Black dots show possible candidates for new cells. Two cases are presented: with condition 2 (left), without condition 2 (right). Arrows show two additional candidates which appear without condition 2.

The example shown in Fig. 5 illustrates how the algorithm of cell choice works. According to condition 1, we consider all cells at the nodes of the grid near the cut (red cells). They are marked with black dots in the figure. Next, we verify that they satisfy condition 2. Some of them may not satisfy it. Fig. 5 (right) contains two additional candidates in comparison with Fig. 5 (left). This small difference appears to be crucial. We will see that condition 2 is necessary for normal regeneration. Finally, among all candidates, which satisfy conditions 1 and 2, we choose the first new cell according to condition 3a or 3b.

**Fig 6:**
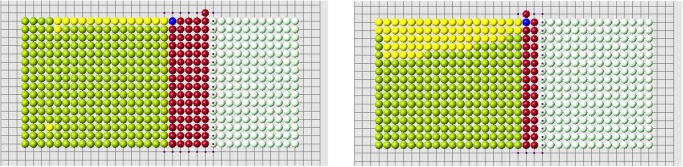
Regeneration without condition 2 and with condition 3a (left) or 3b (right).

Continuation of regeneration without condition 2 is shown in Fig. 6. When the first row is filled, the best cell according to condition 3a is in the middle of the second row. However, condition 3b favors a cell at the continuation of the cut row (the last row of remaining cells). The algorithm with condition 3a (without condition 2) shows a better regeneration. New cells fill 5 rows of the original form and then it adds a cell outside the original form. Yellow cells show the places where condition 2 is not satisfied. The blue is the worst among such cells, that is where the difference $u_{i}-u_{i}^{*}$ is maximal.

**Fig 7:**
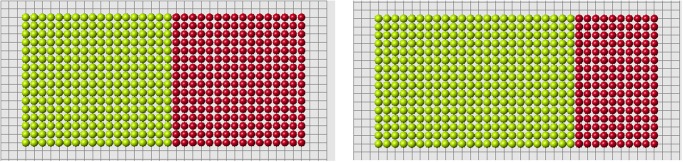
Regeneration with condition 2 and two different amputated parts. Both algorithms 3a and 3b give the same result.

**Fig 8:**
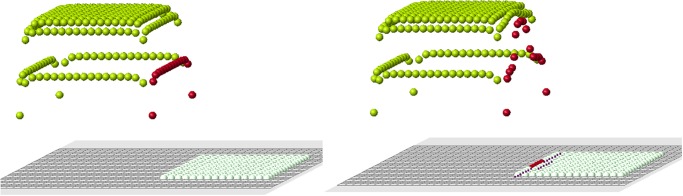
New signal in control cells before regeneration (left) after regeneration of 5 cells (right).

**Fig 9:**
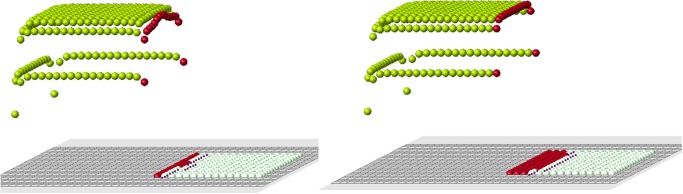
New signal in control cells after regeneration of 20 cells (left) and 60 cells (right).

If we use condition 2, then both algorithms (3a and 3b) correctly regenerate the original form (Fig. 7). Figs. 8 and 9 show the evolution of the new signal *u*_*i*_(*t*) in the control cells during regeneration. It gradually grows and after the regeneration of several dozens of cells it comes close to the old signal.

Let us note that the algorithm of regeneration may not preserve the symmetry of the structure. Indeed, if there are two candidates for new cells which are equivalent with respect to conditions 1–3, we first add one of them. After that the second one may not satisfy these conditions any more.

### Nonlinear diffusion

The signal used above, in order to model regeneration and morphogenesis, corresponds to chemical concentrations or to electric potential. Both of them can be described by the equation

$$\begin{array}{c} \Delta u-bu=h(x), \end{array}$$

where Δ*u* is the Laplace (or diffusion) operator, the second term in the left-hand side of this equation describes consumption or destruction of this signal, *h*(*x*) is a source term. In this case the solution *u*(*x*) decays exponentially for *b* > 0 and as a logarithm for *b* = 0.

In the modelling above, we also considered polynomial decay of the solution. In order to obtain it as a solution of diffusion equation, we need to introduce nonlinear diffusion. Consider the corresponding equation

$$\begin{array}{c} {\left(a\left(u\right)u^{\prime }\right)}^{\prime }-bu=0 \end{array}$$

in the half-axis *x* > 0 with the boundary condition *u*(0) = 1. We look for the solution decaying at infinity. We can reduce this equation to the system of the first-order equations:

$$\begin{array}{c} a\left(u\right)u^{\prime }=p,\;\;\;\;p^{\prime }=bu. \end{array}$$

Then

$$\begin{array}{c} p\frac{dp}{du}=ba\left(u\right)u\;. \end{array}$$

Taking into account the boundary condition *p*(0) = 0, we can integrate the last equation:

$$\begin{array}{c} p^{2}\left(u\right)=2b\int_{0}^{u}a\left(v\right)vdv. \end{array}$$

Set *a*(*u*) = *u* ^−*k*^. Then we get

$$\begin{array}{c} u^{\prime }=u^{k}p\left(u\right)=-\sqrt{\frac{2b}{2-k}}\;u^{1+k/2}\;. \end{array}$$

From this equation and the boundary condition we obtain

$$\begin{array}{c} u\left(x\right)=\frac{1}{{\left(1+cx\right)}^{2/k}}\;,\;\;\;\;c=\frac{k}{2}\;\sqrt{\frac{2b}{2-k}}\;. \end{array}$$

Hence, a solution exists for any *k*, 0 < *k* < 2. It can decay with the rate 1/*x* ^*n*^ with any *n* > 1. It corresponds to the decay rate considered in numerical simulations.

## Results

### Dependence on parameters

The model contains only one physical parameter, the rate of decay of the signal, and one numerical parameter related to verification of condition 2. We consider the function *f*(*d*), which shows how the signal depends on distance, in two different forms:

$$\begin{array}{c} f_{1}\left(d\right)=d^{-n}\;,\;\;\;\;\;\;f_{2}\left(d\right)=e^{-nd}\;, \end{array}$$

where *n* > 0. All results shown in the previous section are obtained with the first function for *n* = 2.

Regeneration depends on the value of parameter *n* and on the size of the domain. In the case of polynomial function *f*_1_(*d*) = *d* ^−*n*^, the best value of this parameter in a relatively narrow range around *n* = 2. If *n* is outside this range, then the size of well regenerated domain decreases.

**Fig 10:**
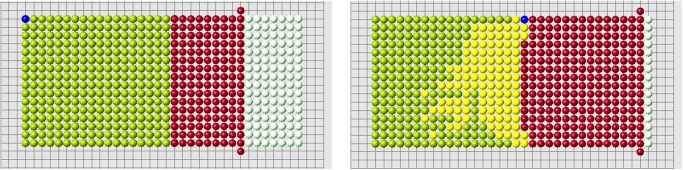
Regeneration with the algorithm 3b and *f*(*d*) = 1/*d* ^*n*^, *n* = 1.6 (left), *n* = 2.4 (right). Red cells show the regenerated domain, white cells its difference with the original domain, blue and yellow cells show where condition 2 is not satisfied.

**Fig 11:**
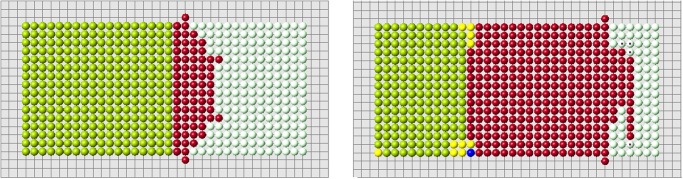
The same as in the previous figure with different values of *n*, *n* = 1 (left), *n* = 2 (right). Possible size of the regenerated domain is essentially greater in the second case but it is also limited.

**Fig 12:**
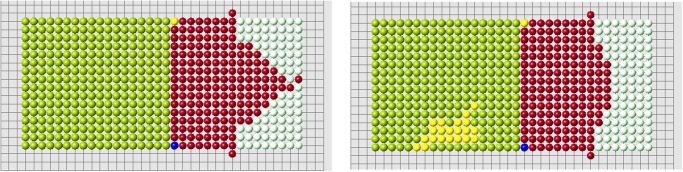
Regeneration with the function *f*(*d*) = exp(−*nd*), *n* = 11 (left), *n* = 12 (right).

Let us note that condition 2 is verified with certain precision. We require that $(u_{i}-u_{i}^{*})/u_{i}^{*}<\epsilon$, where *ϵ* is a small positive number (*ϵ* = 10^−14^ in the simulations shown in Figs. 10–12. If this inequality is not satisfied in any of the control points (cells), then the simulation is stopped. Yellow and blue points show where the condition $u_{i}<u_{i}^{*}$ is not satisfied. Such points can appear during the simulation.

### Other forms

**Fig 13:**
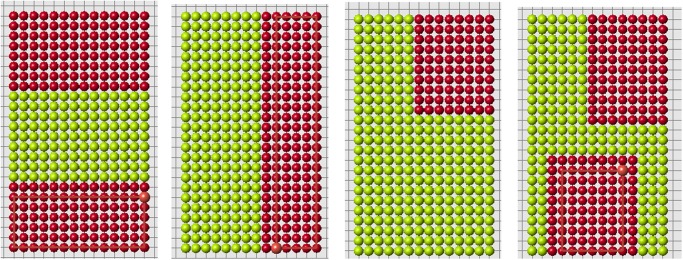
For the same original cell structure we can cut off different parts and regenerate the same original form. Red cells show the regenerated parts.

**Fig 14:**
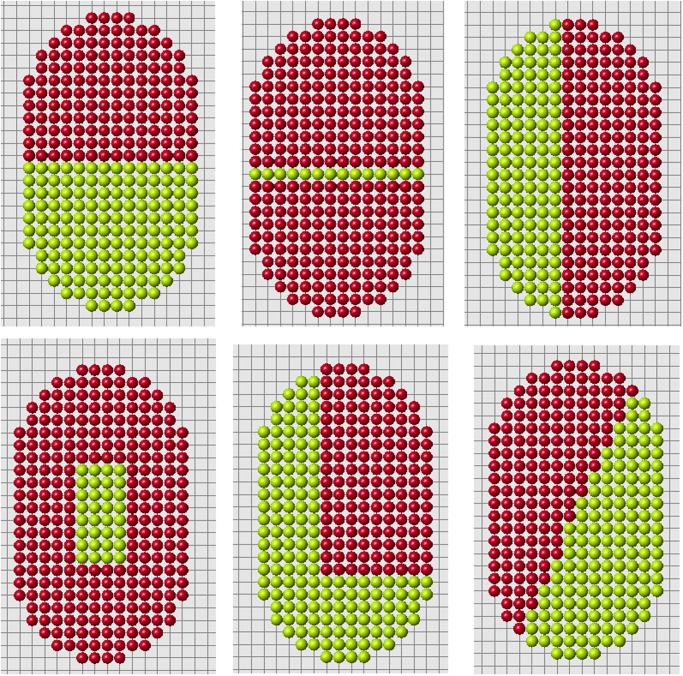
Regeneration of ellipse.

Up to now we considered a model example with a fixed shape of initial and reduced domains. The algorithm presented above allowed us to obtain the exact solution of the problem of regeneration for some class of original and reduced domains. Some examples are shown in Figs. 13 and 14 for rectangular and ellipsoid domains. We can cut off different parts of these domains and the system restores them to the original form.

Let us note that in all examples of correct regeneration, the regenerated part of the domain is convex or it is composed of several separated convex subdomains for which regeneration occurs almost independently. The algorithm described above is not aimed for nonconvex domains because the distance between two cells is measured along the straight interval connecting them. If the domain is not convex and the signal propagates along the tissue, then the distance between two points should also be measured along the tissue.

### Nonconvex domains

**Fig 15:**
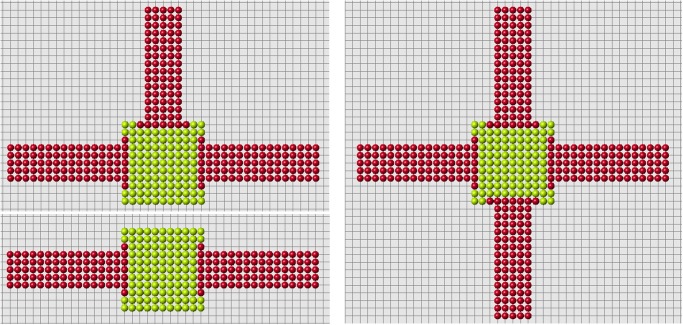
Regeneration of letters. Regenerated part is shown in red, remaining part after amputation in green. The red cells adjacent to green cells belong to the remaining part (blastema).

The regenerated domains presented in the previous sections are not necessarily convex. However, each regenerated part is convex. The signals produced and received by cells decay with distance, which is measured along the straight line connecting cells. In the case where the domain is not convex, the signal can propagate outside of the domain filled by cells. Biologically, this means that there are some other tissues which can transmit signals but which do not participate in the process of regeneration. Some examples of regeneration of nonconvex structures are shown in Fig. 15.

**Fig 16:**
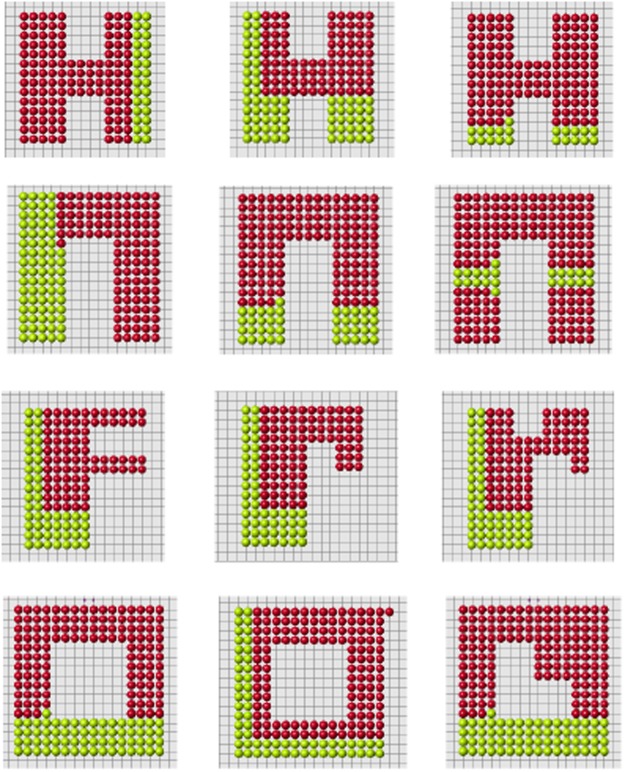
Examples of correct (left and right images) and wrong (middle image) regenerations. A small change in the cell structure modifies the signals received by the control cells *A* and *B*. This can result in the appearance of a wrong cell *C* (see the explanation in the text.

As discussed above, if the size of regenerating domain is too large, the control cells cannot distinguish signals correctly, and regeneration becomes abnormal. This critical size of the domain depends on its form. Moreover if the domain is not convex, it becomes more difficult for control cells to interpret signals and the size of the domain is even more important. Let us illustrate it with the example in Fig. 16. Correct regeneration is shown in the left image. We remove one more row of cells (middle image) and regeneration becomes wrong. We stop the process of regeneration when the first wrong cell appears (it is cell *C* in the middle image). Why does it appear here and not in the left image?

The nearest control cell to the cell *C* in the middle image is the cell *B*. Let $u_{B}^{*}$ be the old signal in cell *B*, and *u*_*B*_(*t*_0_) the new signal just before the appearance of cell *C*. The signal *u*_*BC*_ produced by cell *C* and received by cell *B* adds to the total signal received by cell *B*. It remains smaller than the old signal,

$$\begin{array}{c} u_{B}\left(t_{0}\right)+u_{BC}\leq u_{B}^{*}. \end{array}$$ (2)

**Fig 17:**
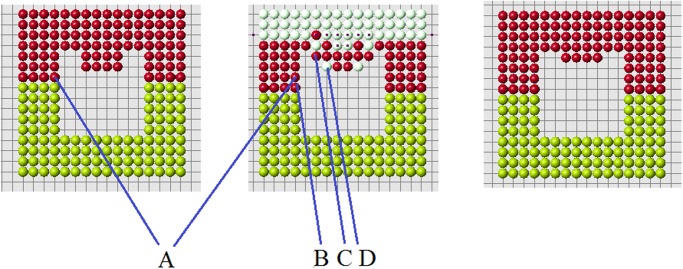
Continuation of the wrong regeneration started in Figure.

Therefore the second condition of the algorithm remains satisfied and cell *C* is added to the regenerated structure. After that regeneration continues incorrectly (Fig. 17).

In the regeneration of the structure shown in Fig. 16 (left), the nearest control cell to cell *C* is cell *A*. Its distance to cell *C* is less than the distance to cell *B*. Therefore, the signal which it receives from cell *C* is greater, *u*_*AC*_ > *u*_*BC*_, and instead of inequality (2) we now have the opposite inequality

$$\begin{array}{c} u_{A}\left(t_{0}\right)+u_{AC}>u_{A}^{*}. \end{array}$$ (3)

Therefore the second condition of the algorithm is not satisfied, cell *C* is not added and regeneration continues in a right way. The structure shown in Fig. 16 (right) also regenerated correctly. The difference in comparison with the structure in the middle image is that it does not contain cell *D* and other cells in the same row. The presence of cell *D* in the original structure increases the old signal $u_{B}^{*}$ received by cell *B*. If this cell is absent, inequality (2) is not satisfied, add cell *C* cannot be added. This provides correct regeneration in the right image.

Thus, the size and organization of the cell structure are important for the correct functioning of the algorithm, especially for nonconvex structures where signals coming from different sides can mask the empty space inside the structure.

## Discussion

We suggest a model of regeneration of cell structures based on cell memory,—implemented as follows. Each cell receives signals from all other cells. Its total value is u*. The cell keeps a memory of this value. If the total signal changes and becomes *u*, the cell produces some substance with the rate proportional to u* − *u*. This substance stimulates the proliferation of new cells. As a result, cell structure grows until the new signal *u* becomes equal to the old signal u*. This mechanism allows cells to implement a kind of means-ends analysis—a working towards regeneration of a specific shape and a cessation of further growth when the correct morphology is achieved a major open question in the understanding of morphostasis in regeneration, remodeling, embryogenesis, and cancer suppression.

The main question is whether this method allows a correct regeneration of cell structure when a part of it is amputated. We show that under some additional conditions which provide continuity of growth and that the new signal cannot exceed the old signal, we can obtain an exact solution of the regeneration problem.

**Limitations of the algorithm.** The algorithm of cell structure regeneration suggested in this work has several limitations. First is the size of the structure. Control cells cannot correctly identify missing cells at a large distance because the signal rapidly decays. However, there is a tradeoff here. If the rate of decay is low, then the boundary of the cell structure is not clearly identified. Added cells will go beyond the original structure and the regenerated shape will be incorrect. On the other hand, rapid decay of the signal also requires high accuracy in the verification of the second condition of the algorithm. If the new signal in some of control cells becomes greater than the old one on a very small value (usually, 10^−7^−10^−15^), then the algorithm is stopped. Biologically this means that control cells are very sensitive. It should be noted however that we consider a qualitative mechanism of regeneration with arbitrary values of parameters, which we cannot relate at the moment to realistic biological parameters.

**Fig 18:**
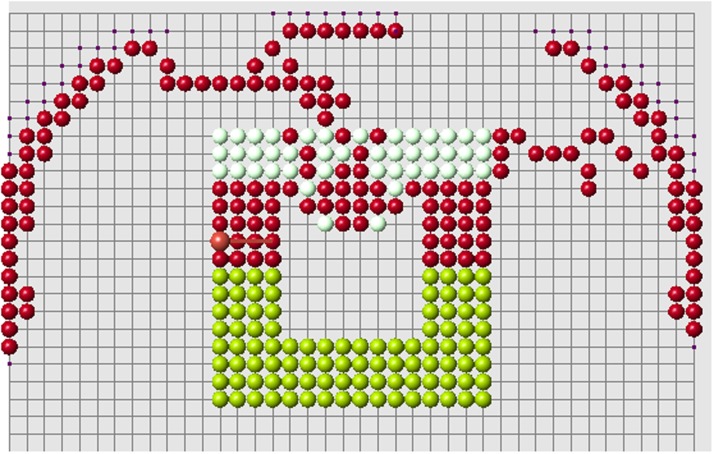
Regeneration with condition 3a and different sizes of the remaining domain (left) or different decay rate *n* of the signal (right). The signal decays as 1/*x* ^*n*^, *n* = 1.5, *ϵ* = 10^−15^.

**Fig 19:**
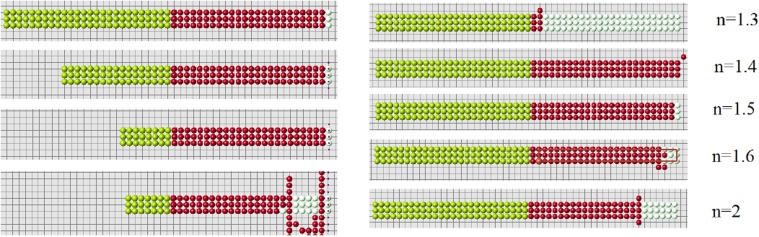
Regeneration with condition 3a and different values of *ϵ* (*n* = 1.5).

Figs. 18 and 19 show regeneration of a long thin domain for different values of parameters and sizes of the domain. There are some optimal values where the algorithm is more efficient. If we consider the size of the cell structure close to its maximal admissible value, the algorithm becomes sensitive to small changes of parameters or geometry. In particular, if the remaining part of the domain after amputation is less than some critical value (Fig. 18, left), then regeneration fails.

Finally, let us recall that the distance between two cells is measured along the straight line connecting them. If the cell structure is not convex, then this interval can be partially outside of this structure. From the biological point of view, this implies existence of other cells (not explicitly included in the model) which fill the empty space and which can transmit the signal but do not participate in the process of regeneration. It is possible to modify the algorithm is such a way that the signal propagates along the structure even in the case where it is not convex. However it then becomes significantly more complex and the path between two points to measure the distance between them can be defined in different ways. This will be explored in subsequent work.

**Reaction-diffusion patterns or cell memory?** Reaction-diffusion systems of equations can describe emergence of patterns. The mechanism of pattern formation mostly used in morphogenesis and regeneration is based on Turing structures. In order to describe emergence of patterns, the reaction-diffusion system should contain at least two equations and should satisfy some additional conditions. They are formulated by H. Meinhardt as short range activation—long range inhibition. Emergence of patterns is based on the interaction of these two substances (Turing called them morphogens). Since these patterns can represent stable stationary solutions, then the perturbed pattern can return to its original form after some time. This is the main idea of regeneration models with reaction-diffusion equations.

Some animals, like planarians or hydra, can regenerate their head or tail (feet) or both. These two organs can produce various signals which propagate in other tissues. If these signals interact with each other (activation-inhibition) and if this interaction results in their nonuniform distribution (pattern formation), then it is possible to imagine that they also participate in the regeneration of the lost organ.

The method suggested in this work does not imply the interaction of two (or more) signals. In the minimal configuration it is sufficient to have only one signal. The main assumption of the model is that cells can keep information about the previous value of this signal when it is changed (< 64 bits of information). One testable prediction of such models is that mechanisms that underlie memory in the nervous system may be likewise implicated in regenerative pattern control.

Let us note that regeneration with memory cells acts locally. It does not require interaction of signals from distant organs. Wound healing is an example of regeneration which occurs locally and where only one tissue can be involved. The model based on cell memory suggests the same approach to wound healing and to regeneration of a single organ or several organs simultaneously.

Interestingly, some species such as planaria possess high morphological plasticity. For example, it is possible to create a two-headed animal from a normal one by perturbing the physiological circuit that normally determines anterior-posterior polarity [30, 29]. This stability of a radically new target morphology (2 head shape, which continues to recur upon future rounds of amputation with no further experimental treatments) does not require genomic modifications. The old one-headed and the new two-headed animals have the same genome. Moreover the new head can be grown anywhere on the body. If one of the two heads or both of them are removed, they regenerate in the same way as they were before. Thus, two-headed planarian regenerates in a two-headed planarian with the same head locations.

It is interesting to compare how different models can regenerate a two-headed planarian. Denote by *D*_1_ the cell structure which corresponds to one-headed planarian and by *D*_2_ the two-headed one. After head amputation in the former, the corresponding domain is denoted by *D*. It is possible to amputate both heads of the two-headed animal reducing domain *D*_2_ to the same domain *D*. Hence we have the same cell structure *D* from which we should be able to regenerate either one-headed or two-headed planarian.

Suppose that regeneration is based on emergence of patterns. Then there are two signals *u* and *v* which remain in the domain *D* after head amputation. Their interaction can produce some nonhomogeneous distribution of these substances by the mechanism of Turing structures. Since the characteristic diffusion time is much less (hours) than time of regeneration (days), we can reasonably assume that they will converge to some stationary distributions in the domain *D* before it begins to grow due to regeneration. If this stationary distribution is unique, then only a unique structure can regenerate and not two different structures with one and two heads. However, it is known that Turing structures in the same domain and for the same values of parameters can be nonunique. If there are two different structures possible in the domain *D*, then one of them can correspond to the one-headed animal while another one to the two-headed planarian. Convergence to these stationary solutions depends on the initial conditions. Since the distributions of *u* and *v* in *D* after amputation from *D*_1_ can differ from those after amputation from *D*_2_, then we can suppose that they will converge to two different stationary solutions. Thus, up to now, this approach admits regeneration of two different structures *D*_1_ and *D*_2_ from the same reduced structure *D*.

A limitation of this approach is that position of the second head can be arbitrary in some range of locations. If each head position should correspond to a different stationary solution in the same domain *D*, then we need to have possibly many such solutions or even a continuous family of such solutions. It is possible to have several different Turing structures in the same domain but if there are several dozens, then it becomes exotic from the modelling point of view and unrealistic biologically. A continuous family of such solutions is not possible even mathematically unless the domain possesses some special symmetry.

**Fig 20:**
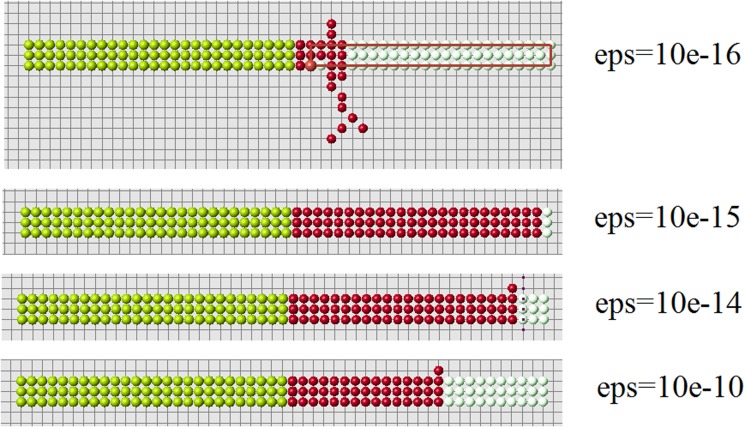
The same reduced part (upper left) can regenerate different original forms (upper right and lower figure).

These arguments should not be considered as a proof of impossibility of this mechanism of planarian regeneration. Such complex and poorly understood processes always leave a possibility for different modelling approaches. On the other hand, the method with cell memory eagerly reproduces two-headed or multi-headed structures with any head location (Fig. 20).

**Morphogenesis.** The model of regeneration discussed above is based on the assumption that cells can register a signal coming from other cells. When a part of the tissue is amputated, the signal is changed. The process of regeneration consists in restoring the tissue which has the same distribution of signals.

A similar approach can be used to describe initial tissue growth. Consider a domain filled by cells. Suppose that each cell has some value $u_{i}^{*}$ prescribed to it. This hypothetical signal appears in the process of embryogenesis due to some genetic and epigenetic factors. It can correspond to distribution of some morphogenes. This initial domain can be considered as an organizing center. The signal distributed inside it stimulates appearance of new cells around it. These new cells will be placed in such a way, that the signal which they produce coincide with $u_{i}^{*}$ inside the organizing center.

**Fig 21:**
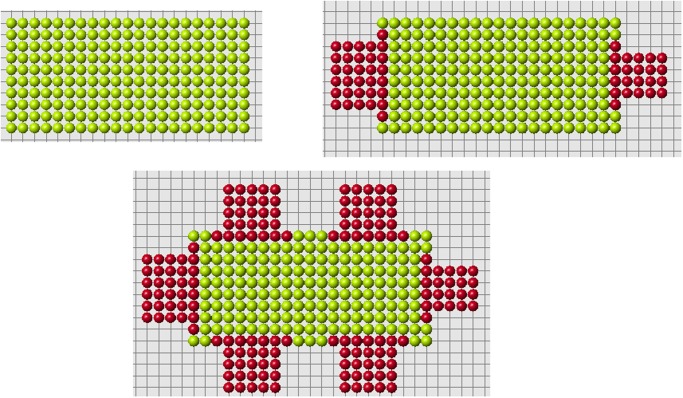
Depending on the signal distribution inside the central square domain, two, three or four red rectangles will grow around it.

An illustration of this mechanism is shown in Fig. 21. The initial domain is a square at the center. Depending on the distribution of the signal inside this domain, different structures can grow from it.

**Morphogenesis versus regeneration.** The speed of animal regeneration can be greater than the speed of its natural growth. If a 7-day old tadpoles tail is cut and allowed to regenerate for 7 more days, then new tail produced will be appropriate in size for a 14-day sized animal. It shows that remaining cells remember the size of the animal before amputation. Moreover, this is also an argument against the pattern formation mechanism. If growth is completely determined by the actual cell structure, then natural growth and growth after amputation would be the same if they start from the same cell configuration.

**Target morphology.** The concept of target morphology is suggested in [5, 16, 17]. The model developed in this work interprets this idea in terms of signals. Let us recall the main assumption of the model. Control cells keep the information about the old signals. At the same time they receive new signals from the growing tissue. Target morphology is the morphology for which the distribution of new signals coincides with the distribution of old signals. In the case of morphogenesis, instead of old signals, there is some given distribution which appears during embryonic development. As before, target morphology is the morphology for which the distribution of received signals coincides with the given distribution. Our conjecture is that generically the target morphology is unique, that is there exists a unique solution of the problem of minimization of signal difference.

**Further developments.** If regeneration is based on cell memory in the remaining tissue, then the process of regeneration can be modified if this memory is modified. Future work in our group will test this model using reagents known to modify cellular memories in the nervous system, applied to regeneration assays.

There are many possible developments of the model. Among them introduction of different cell types (differentiation) and the model of long distance regeneration. Both of them will require introduction of additional signals. At further development of this model, we are considering adding biological cells as distributed objects (not as mathematical points) and associating signals with the points at the surface of their exterior membrane, to more closely tie the modeling dynamics with known biophysical or molecular components of cellular function. Altogether, development of models such as this one, which show unambiguously (algorithmically) how shape is preserved and repaired, are an essential counterpart to genetic and biophysical data. Together, the integration of constructivist modeling and molecular biology will enable biomedical control of not only gene expression but of large-scale anatomical outcomes.
